# Case report: Small bowel obstruction secondary to congenital transmesenteric internal hernia in a cat

**DOI:** 10.3389/fvets.2024.1358797

**Published:** 2024-04-23

**Authors:** Min-Hee Kang, Young-Chil Kang, Jun-Won Yoon, Hee-Myung Park

**Affiliations:** ^1^Department of Bio-Animal Health, Jangan University, Gyeonggi-do, Republic of Korea; ^2^Animal Hospital with Love, Seoul, Republic of Korea; ^3^Department of Veterinary Internal Medicine, College of Veterinary Medicine, Konkuk University, Seoul, Republic of Korea

**Keywords:** feline, gastrointestinal disease, internal hernia, congenital disease, mesenteric defect

## Abstract

An 8-month-old castrated male British Shorthair cat presented with acute anorexia and vomiting. The overall clinical presentation included generalized depression. Physical examination revealed palpable abdominal mass, thus foreign body or intussusception was suspected. Abdominal radiographs showed segmental dilation of small intestine and ultrasonography revealed target lesion with dilated small bowel loops and disrupted normal wall layering, suggestive of intussusception. Exploratory laparotomy confirmed congenital mesenteric defects associated with small intestinal obstruction. Surgical intervention involved dissection, ligation of encircling blood vessels, and closure of mesenteric defects. The cat was discharged after 3 days, exhibiting normal postoperative recovery. To our knowledge, this is the first case report of congenital mesenteric defect associated with small intestinal obstruction in a cat. While internal hernias are rare, it is essential to include them in the differential diagnosis for cases of intestinal obstruction, particularly in patients with no history of previous surgery or trauma. The potential for strangulation and ischemia in the affected loops elevates internal hernias to a critical, life-threatening condition, emphasizing the need for prompt recognition and urgent surgical intervention as an emergency.

## Introduction

Internal hernia (IH) is defined by the protrusion of abdominal organs through a peritoneal or mesenteric defects into a compartment within the abdominal cavity (1, 2). IH is a rare condition with a prevalence of less than 1% in the general population and is primarily reported in children as a congenital anomaly (3). In adults, it may develop as an acquired condition resulting from abdominal injury, peritoneal inflammation, or iatrogenic factors following previous surgical procedures (2, 4). As previously noted, IH can lead to intestinal obstruction in humans. When intestinal obstruction occurs, the affected intestinal segment undergoes strangulation, resulting in ischemia and posing a life-threatening risk (5, 6). Therefore, timely diagnosis is crucial; however, the identification of IH still remains a challenge in humans (4, 6). IH has not been previously reported in the field of veterinary medicine.

This report describes a rare cause of small intestinal obstruction associated with internal hernia in a cat. The present report describes congenital mesenteric defect for the first time in veterinary medicine.

## Case description

### Case presentation and diagnostic investigations

An 8-month-old male castrated British Shorthair cat, weighing 3.8 kg, was admitted to the emergency service for acute abdomen accompanied by 1 day history of anorexia and vomiting. The cat received all necessary vaccinations and deworming, lived indoors alone, and had no recent history of illness. On presentation, the cat was generally depressed and reluctant to stand. Physical examination revealed mild abdominal distension and discomfort. Abdominal palpation revealed palpable mass located in the right abdominal quadrant. Other physical examination was unremarkable.

**Figure 1:**
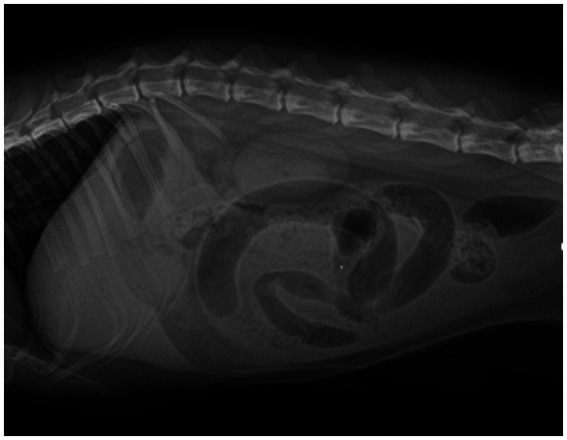
Lateral abdominal radiograph of an 8-month-old British short hair cat with acute abdomen. Preoperative right lateral abdominal radiograph of this cat showed gas-filled segmental dilation of small intestinal loops, with no apparent presence of foreign bodies or mass lesions.

**Figure 2:**
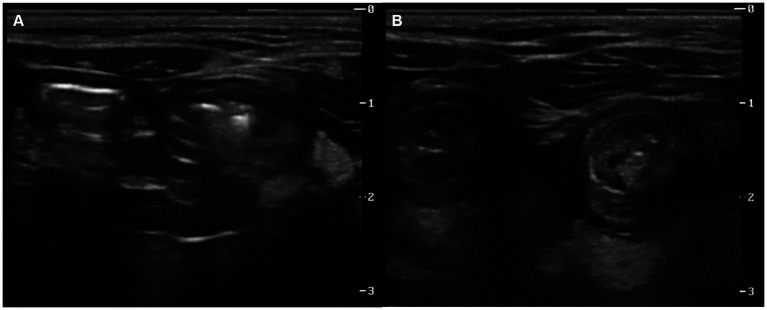
Ultrasonography images of an 8-month-old British short hair cat suspected with a small intestinal mechanical obstruction. Longitudinal **(A)** and transverse **(B)** images at the level of palpable mass-like lesion revealed dilated loops of the small intestine. Marked segmental dilation of intestinal loops with reduced peristaltic movement of the intestinal contents was observed. The normal wall layering of the intestine was disrupted, and on the transverse image **(B)**, the lesions resembled multi-layered concentric rings. Hyperechoic fat adjacent to the lesion was also detected.

Serum biochemistry and complete blood count revealed an elevation in platelet counts (1,231 × 10^9^/L, reference 300–800 × 10^9^/L) and mild hyperglycemia (173 mg/dL, reference 53–150 mg/dL). Feline serum amyloid A was also mildly elevated (29 μg/mL, reference 0–10 μg/mL). For the assessment of abdominal mass-like lesions, additional imaging evaluations were conducted using radiographs and ultrasonography. Thoracic radiographs were unremarkable. Abdominal radiographs showed segmental gas-filled dilation of the small intestine (Figure 1). Abdominal ultrasound revealed significant segmental dilation of small intestinal loops, with disruption of normal layering in the intestinal wall (Figure 2). The movement of the intestinal contents at the dilated lesions was decreased. No visible foreign body or mass was detected; however, the transverse image of the lesions exhibited characteristics resembling a target lesion (Figure 2B), suggesting a high suspicion of small intestinal intussusception.

Based on these findings, mechanical obstruction due to small intestinal intussusception was strongly suspected in this cat. Therefore, explorative laparotomy was recommended for both diagnosis and treatment.

**Figure 3:**
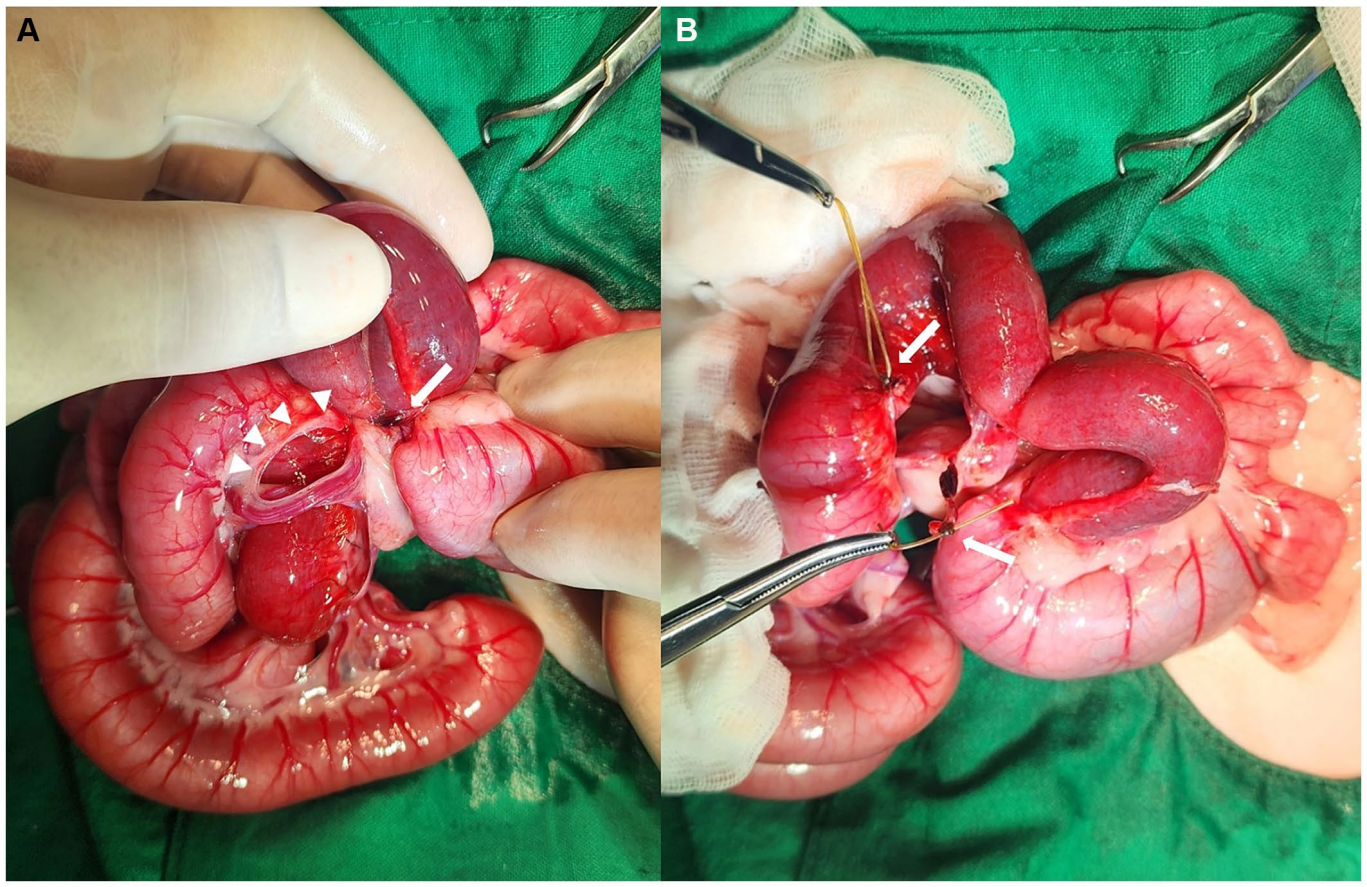
Intraoperative images of an 8-month-old British short hair cat with a transmesenteric internal hernia. Emergency laparotomy revealed congenital mesenteric defects (**A**, arrow heads) with the engorged vessels encircling the mid-jejunum (**A**, arrow). The blood vessels around the small intestines were carefully isolated and ligated (**B**, arrows). The aboral part of the jejunum showed mild distension and hyperemia; fortunately, no necrotic areas were identified.

**Figure 4:**
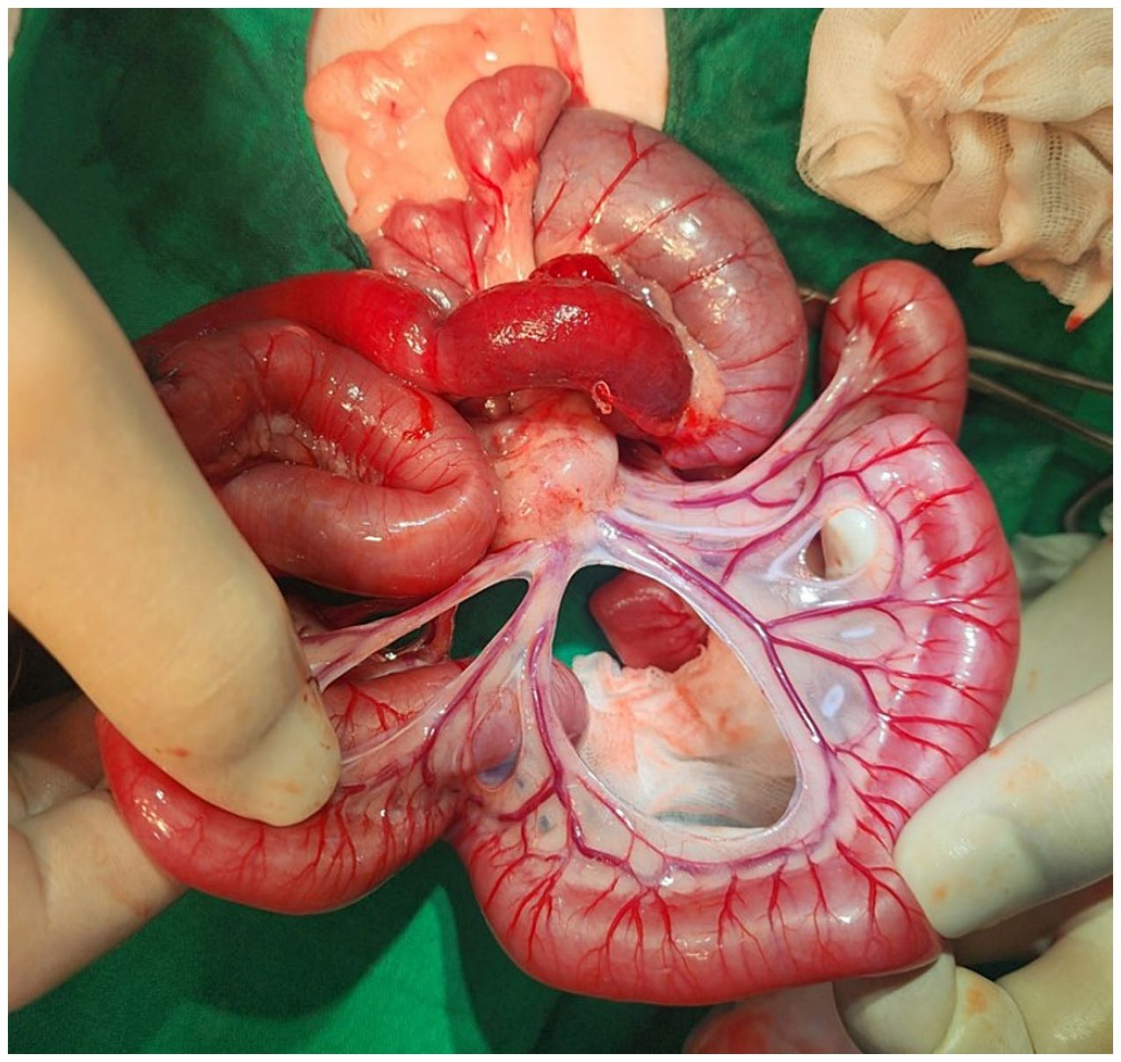
Gross images of an 8-month-old British short hair cat with a congenital small bowel mesenteric defect. Congenial defects of the mesentery with internal hernia were confirmed in this cat. When the jejunum was exposed, the mesenteric vascular arcades include *vasa recta* appeared normal, but a significant portion of the mesentery were absent.

Preoperative stabilization was initiated with flow-by oxygen, amoxycillin/clavulanic acid injection (20 mg/kg, SC, Kuhnil, South Korea) and fluid therapy with Hartmann’s solution (JW pharmaceutical, South Korea). Anesthesia was induced with tiletamine-zolazepam (5 mg/kg, IM, Virbac, Carros, France) and anesthesia was maintained with 1% isoflurane. A standard ventral midline laparotomy was performed, exposing distended small intestinal loops. The obstruction site involved a herniated small intestine that was strangulated by the surrounding mesenteric blood vessels (Figure 3A). A substantial portion of the small intestinal mesentery were absent, although the mesenteric vascular arcades include *vasa recta,* appeared normal (Figure 4). The blood vessels encircling the segments of the small intestine were carefully dissected and ligated, enabling the observation of the intestinal segments (Figure 3B). Dilatation and congestion of the intestine proximal to the obstruction were identified. Since there were no signs indicating intestinal ischemia or necrosis, resection and anastomosis surgery were not conducted. After confirming the normal movement of intestinal contents, large mesenteric defects were sutured with simple interrupted pattern sutures using 4–0 monofilament absorbable polyglyconate (Maxon, Covidien, Dublin, Ireland). Congenial defects of the mesentery with internal hernia were confirmed in this cat. The cat made a satisfactory recovery from surgery. Intravenous fluids and antibiotic therapy (amoxicillin/clavulanic acid, 20 mg/kg, SC, q 12 h) was continued. Maropitant citrate (1 mg/kg, SC, q 24 h; Zoetis, Gerona, Spain) was used for antiemetic, and gabapentin (10 mg/kg, PO, q 8 h, Pfizer, Freiburg, Germany) was provided for pain control. The cat showed no signs of vomiting, and its appetite returned to normal 2 days after surgery. Discharge was made 3 days after the surgery, and oral medications, including antibiotics, antiemetics, and pain relief, were continued for 7 days. A follow-up visit was scheduled for suture removal and re-evaluation after 10 days. The cat’s overall condition was normal, and there were no observed abnormal intestinal signs.

## Discussion

Internal hernia (IH) is a rare condition in humans and remains unreported in dogs and cats. Its incidence is estimated to range from 0.2 to 0.9% in autopsy cases, contributing to 0.6–5.8% of all instances of small bowel obstruction (SBO) (4, 6). IH can lead to complications such as intestinal obstruction, strangulation, and subsequent necrosis affecting varying lengths of the intestine (4).

This case report describes a rare congenital defect in the small intestinal mesentery that resulted in SBO in a cat. The cat presented with acute digestive signs, and a physical examination identified an intra-abdominal mass-like lesion. Considering the cat’s age and medical history, the primary differentials included foreign body, intussusception, or other cause of obstruction.

The predominant reported etiologies of gastrointestinal obstruction in dogs and cats include foreign bodies, intussusception, neoplasia, abscess, granulomas, and the formation of strictures (7). Among the various etiology, ingestion of foreign bodies and intussusception was the most common cause of mechanical obstruction in cats (8). When intestinal obstruction occurs due to any cause, it results in reduced intestinal contents and inhibited intestinal motility. Furthermore, direct distension of the intestinal wall by foreign object can induce venous congestion and edema, potentially leading to more severe vascular damage and necrosis (9). For a favorable prognosis, prompt diagnosis and therapeutic intervention was essential.

In this case, both radiographic and ultrasound examinations was used for diagnosis. Although abdominal radiography suggested the potential for intestinal obstruction, but identifying the exact cause was not possible. Subsequent ultrasound examination raised suspicion of intestinal intussusception. As a result, a surgical approach was conducted in this cat, leading to the ultimate diagnosis of intestinal obstruction due to IH.

Internal hernias are classified based on the location of the defect openings, with Meyers categorizing them into paraduodenal, pericecal, foramen of Winslow, transmesenteric, transmesocolic, intersigmoid, and retroanastomotic types (10). Among these, transmesenteric herniation caused by a congenital anomaly is the most common type and eight congenital cases were first reported in 1836 (11). The pathophysiology of mesenteric defects remains unclear, with various hypotheses proposed, including partial regression of the dorsal mesentery, developmental enlargement of a poorly vascularized areas, rapid elongation of a mesenteric segment, and compression of the mesentery by the colon during the in prenatal period (4, 12). Congenital mesenteric defects are frequently associated with other gastrointestinal anomalies, particularly small bowel atresia (13).

With recent advancements in diagnostic imaging technology, the characterization of different types of IH using CT has been on the rise (4). However, preoperative diagnosis of mesenteric defect still remains challenging for both clinicians and radiologist (6).

In this report, limitations arose as additional CT scans could not be conducted. However, a rapid diagnostic approach and exploratory laparotomy successfully averted further complications. The segment of bowel affected by obstruction in this cat was relatively short, particularly when compared to the extensive mesenteric defect site. Additionally, the duration of ischemia was not prolonged, resulting in minimal intestinal damage and a quick recovery. Histopathological examination of the mesentery was not conducted in this case, although it could have provided valuable insights into the congenital etiology of the condition and the nature of tissue changes. Generally, diagnoses are typically made through emergency exploratory laparotomy, and histopathological information is rarely available even in human cases. Transmesenteric hernia is a rare cause contributing to small bowel obstruction. In the case of bowel obstruction in relatively young animals, it is advisable to consider congenital mesenteric defect as a potential differential diagnosis.

In conclusion, to the best of our knowledge, this case report represents the first documentation of a small intestinal mesenteric defect in a cat. The consideration of a congenital mesenteric defect should be included in the differential diagnosis for relatively young dogs and cats displaying signs of SBO, particularly in the absence of external hernia, suspicion of a foreign body, prior abdominal surgery, or trauma.

## Data availability statement

The raw data supporting the conclusions of this article will be made available by the authors, without undue reservation.

## Ethics statement

Ethical approval was not required for the studies involving animals in accordance with the local legislation and institutional requirements because This paper is a case report based on client-owned animals, and consent was obtained from the owner of the cat for the publication of this case report and any accompanying images. Written informed consent was obtained from the owners for the participation of their animals in this study.

## Author contributions

M-HK: Conceptualization, Writing – original draft, Writing – review & editing. Y-CK: Data curation, Writing – original draft, Writing – review & editing. J-WY: Data curation, Writing – original draft, Writing – review & editing. H-MP: Supervision, Writing – original draft, Writing – review & editing.
